# Phosphorus Concentration in Knee Joint Structures of Patients Following Replacement Surgery

**DOI:** 10.3390/ijerph16040525

**Published:** 2019-02-13

**Authors:** Ciosek Żaneta, Kosik-Bogacka Danuta, Łanocha-Arendarczyk Natalia, Kot Karolina, Karaczun Maciej, Ziętek Paweł, Kupnicka Patrycja, Szylińska Aleksandra, Bosiacki Mateusz, Rotter Iwona

**Affiliations:** 1 Departament of Biology and Medical Parasitology, Pomeranian Medical University in Szczecin, Powstańców Wielkopolskich 72, 70-111 Szczecin, Poland; nlanocha@pum.edu.pl (Ł.-A.N.); kotkarolina17@gmail.com (K.K.); 2 Department of Medical Rehabilitation and Clinical Physiotherapy, Pomeranian Medical University in Szczecin, Żołnierska 54, 71-210 Szczecin, Poland; aleksandra.szylinska@gmail.com (S.A.); iwrot@wp.pl (R.I.); 3 Independent of Pharmaceutical Botany, Department of Biology and Medical Parasitology, Pomeranian Medical University in Szczecin, Powstańców Wielkopolskich 72, 70-111 Szczecin, Poland; kodan@pum.edu.pl; 4 Chair and Clinic of Orthopaedics, Traumatology and Oncology, Pomeranian Medical University, Unii Lubelskiej 1,71-252 Szczecin, Poland; karaczun@orthomail.com (K.M.); paulz@wp.pl (Z.P.); 5 Department of Biochemistry and Medical Chemistry, Pomeranian Medical University, Powstańców Wielkopolskich 72, 70-111 Szczecin, Poland; pkupnicka@gmail.com; 6 Department of Functional Diagnostics and Physical Medicine, Pomeranian Medical University in Szczecin, Żołnierska 54, 71-210 Szczecin, Poland; bosiacki.m@gmail.com

**Keywords:** phosphorus, Hoffa’s fat pad, endoprosthesia

## Abstract

The aim of the study was to assess phosphorus (P) concentration in structures of the knee joint—including the tibial spongy bone, articular cartilage, meniscus, anterior cruciate ligament, and infrapatellar fat pad (Hoffa’s fat pad)—of patients following knee joint replacement. The study also aimed to assess the influence of selected biological and environmental factors on P concentration in studied parts of the knee joint. Phosphorus concentration was determined using inductively coupled plasma-atomic emission spectrometry (ICP-AES). Statistically significant differences in P concentration were found between different elements of the knee joint. The highest P concentration was measured in the spongy bone (72,746.68 mg kg^−1^ dw) and the lowest in the Hoffa’s fat pad (1203.19 mg kg^−1^ dw). P levels were unaffected by gender, age, BMI, place of residence, smoking, or alcohol consumption. Data on P concentration in the osteoarticular elements of the knee may be useful in the interpretation and evaluation of biochemical, morphological, and mechanical changes occurring in the body.

## 1. Introduction

Bone tissue is an important biological medium in the assessment of environmental exposure to various elements, including toxins. Due to the slow nature of bone remodeling, concentrations of elements in bone reflect their long-term accumulation in the body. Prior studies have demonstrated that element levels are influenced by biological and environmental factors, including gender, age, diet, place of residence, smoking, and alcohol consumption.

The knee joint is the largest joint in the human body, and the most prone to damage. Untreated knee injuries, gender, age, obesity and smoking predispose people to faster degeneration and abrasion of joint cartilage, accelerating loss of joint function and development of osteoarthitis [1]. According to a report by the World Health Organization, gonarthrosis is the world’s fourth most common cause of disability among women and the eighth most common among men [2]. Additionally, osteoarthritis is the most common indicator for knee joint arthroplasty procedures. With the substantial increase in the prevalence of osteoarthritis—and the resulting need for knee joint alloplasties—in the last century, investigation of causal factors and possible therapeutic targets for the disease is of great interest [3].

Phosphorus (P) is a macroelement with roles in numerous processes in the body. It is a major component of bone tissue, its main structural element after calcium (Ca). Phosphorus accounts for 0.65–1.1% of body weight in adults, of which 85% is deposited in bone tissue in the form of hydroxyapatite and 15% is found in soft tissues [4]. Proper saturation of bones with calcium and phosphorus salts determines their mechanical resistance. The amount of P in a healthy human body weighing around 70 kg is 700–800 g, while in serum it ranges from 0.84 to 1.45 mmol/L [5]. Abnormalities resulting from P deficiency or other metabolic disorders may significantly affect bone mineralization. Studies have also shown that excessive P intake may lead to increased loss of bone mass and deterioration of biomechanical properties and, if additionally associated with low Ca supply, may affect hormonal regulation of calcium-phosphate balance and vitamin D synthesis [6]. The ratio of Ca to P in the diet is an important factor in ensuring the proper course of bone formation and should be 1:1 (molar) or 1.3:1 (in weight units) [7,8]. The main dietary sources of organic P are protein-rich plant and animal products, including dairy products, meat, pulses, nuts, chocolate, and mushrooms [9]. Phosphorus is absorbed in the gastrointestinal tract and through the skin, while its vapors are absorbed by the respiratory tract [10]. About 65% of the P in food is absorbed by the small intenstine [11].

Due to its importance in bone tissues, research has begun to examine a potential role for P in the development of osteoarthritis; however, thus far, results are few. In light of the lack of existing data on P concentration in knee joint structures, this study endeavored to assess the concentration of this element in the tibial spongy bone, joint cartilage, meniscus, anterior cruciate ligament, and infrapatellar fat pad (Hoffa’s fat pad) of patients following knee replacement surgery, as well as to assess the influence of selected biological and environmental factors on P concentration in studied parts of the knee joint.

## 2. Materials and Methods

The research was conducted between 2014–2017 and was approved by the Bioethics Commission of the Pomeranian Medical University in Szczecin (resolution no. KB-0012/56/14).

The study group consisted of women (*n* = 34) aged 56 to 87 (mean age 73.1 ± 8.4 years) and men (*n* = 12) aged 59 to 85 (mean age 73.6 ± 8.7 years) from the Zachodniopomorskie (West Pomeranian) Voivodeship. Patients were hospitalized in the Department and Clinic of Orthopedics, Traumatology and Oncology of the Motor System at the Pomeranian Medical University in Szczecin. All patients underwent knee joint replacement with reconstruction of the intercondylar area using an autologous bone graft. During knee joint arthroplasty, an intramedullary device is used for the femur. A hole is filled with an autologous bone graft, primarily to stop bleeding from the medullar cavity, restore tissue continuity, and strengthen the intercondylar area. Each patient received a full explanation of the nature and purpose of the study and agreed in writing to participate. Patients were given the opportunity to withdraw at any stage of the examination. In order to homogenize the patient group, patients falling under the following criteria were excluded: aged under 55 years and with history of work in heavy industry in the period of 20 years prior to the study. Indication for knee replacement surgery in the examined patients was left or right knee osteoarthritis (26 and 20 patients, respectively) resulting from basic disease, such as psoriatic arthritis (*n* = 1) and knee trauma (*n* = 1).

Before surgery, information was obtained from each patient concerning health status and environmental exposure including place of residence, smoking, and alcohol consumption. A standard physical examination with measurements of weight and height was carried out, with subsequent determination of Body Mass Index (BMI). According to WHO criteria, values of 18.5–24.99 kg/m^2^ were assumed to be in the normal BMI range, while the values for underweight, overweight, I, II, and III degree (huge) obesity were respectively: <18.5; 25–29.99; 30.0–34.9; 35.0–39.9; and ≥40.0 kg/m^2^. Before surgery, each patient underwent a diagnostic examination, including X-ray and/or magnetic resonance imaging (MRI) of the knee joint. In all patients, there were no post-operative complications.

The following tissues were sampled during knee joint replacement surgery for further analysis: tibial spongy bone (*n* = 46), joint cartilage (*n* = 46), meniscus (*n* = 46), anterior cruciate ligament (*n* = 46), and infrapatellar fat pad (*n* = 46). In all cases, the tissue samples collected were operational waste and are routinely disposed of during the procedure. The material was obtained sterilely and the samples, marked with code numbers, were stored in sterile containers at −27 °C.

The material was weighed with an analytical balance Sartorius YPD03-OCE (Sartorius AG, Germany) to an accuracy of 0.0001 g, and then dried to constant weight at 105 °C. The weight of the samples was in the range from 0.5500 to 0.6500 mg. Dried samples were crushed in an agate mortar. The samples were subjected to microwave mineralization using the MARS 5, CEM system. Atomic emission spectrometry with excitation in inductively coupled plasma (ICP-AES, ICAP 7400 Duo, Thermo Scientific) was used to assess P concentration. Analytical procedure was tested by determination of the tested element in standard reference material NIST 1486 Bone Meal. Average recovery of the P was 107.5%.

Statistical analysis was performed using Statistica 10.0 software (StatSoft, Inc. Tulsa, OK, USA). Normality of the distribution of phosphorus concentrations was assessed by Shapiro-Wilk test. The concentration of the element was expressed using arithmetic mean (AM), standard deviation (SD), median (med.), and range (min–max). Assessment relationship of P concentration in the majority of knee joint elements was performed using the Spearman rank correlation. Comparison of P concentration according to gender, BMI category, smoking, and alcohol consumption was performed by Mann-Whitney or Kruskal-Wallis tests, depending on the number of compared groups. Differences were assumed to be statistically significant at *p* < 0.05.

## 3. Results

The highest mean P concentration was found in the spongy bone (72,746.68 mg kg^−1^ dw) and the lowest was found in the Hoffa’s fat pad (1203.19 mg kg^−1^ dw).

Table 1 presents the P concentration relationship between the majority of knee joint elements.

There was a positive correlation in phosphorus concentration between spongy bone and infrapatellar fat pad (*r* = 0.293, *p* = 0.048); meniscus and anterior cruciate ligament (*r* = 0.398, *p* = 0.006). We observed negative correlation in P concentration between joint cartilage and meniscus (*r* = −0.386, *p* = 0.008).

In the spongy bone, the highest P concentration was recorded in overweight patients- a man aged 81 years (98,448.56 mg kg^−1^ dw) and a woman aged 83 years (97,343.44 mg kg^−1^ dw). Woman had previously undergone left and right hip joint replacement in 2007. The lowest P concentration was measured in the spongy bone of a 79-year-old man (51,266.70 mg kg^−1^ dw) with diagnosed arterial hypertension, Parkinson’s disease, pulmonary embolism, and suspected Graves’ disease.

In cartilage, the highest P concentration was recorded in two female subjects (~100,000 mg kg^−1^ dw). One of the patients presented with arterial hypertension, first degree obesity, and diabetes, while the second patient had previously undergone arthroplasty of the other (right) knee joint due to osteoarthritis. The lowest cartilage P concentration (39,067.57 mg kg^−1^ dw) was reported in a post-ischemic stroke woman with first degree obesity.

In the meniscus, the highest P concentration (33,893.79 mg kg^−1^ dw) was observed in the 79-year-old patient who displayed the lowest P in the spongy bone. The lowest observed P concentration in the meniscus (472.85 mg kg^−1^ dw) was in a 75-year-old woman with arterial hypertension and first degree obesity. Low meniscus P levels were also observed in a 68-year-old woman with osteoporosis, kidney stones, and previous arthroplasty of the right knee joint. In this patient, the concentration of P in the meniscus did not exceed 480 mg kg^−1^ dw.

In the ACL, the highest concentration of P was observed in an 80-year-old woman with diabetes, hypertension, and ischemic heart disease. The P concentration exceeded 90,000 mg kg^−1^ dw. The lowest P concentration in the ACL (562.70 mg kg^−1^ dw) was measured in a 77-year-old obese woman post-myocardial infarction, with hypertension, hypothyroidism, and prior right knee joint arthroplasty.

In the Hoffa’s fat pad, the highest P concentration (10,325.85 mg kg^−1^ dw) was observed in an 85-year-old man with previous hip replacement and diagnosed arterial hypertension. The lowest concentrations of P were recorded from a 67-year-old obese woman (235.11 mg kg^−1^ dw) and a 71-year-old woman with arterial hypertension and first degree obesity (247.99 mg kg^−1^ dw). Previously, both patients had undergone arthroplasty of the right knee joint.

Table 2 presents measured P concentrations in knee joint elements as they relate to certain biological factors, including gender, age, and BMI of patients. In terms of gender of the examined patients, in women, the highest concentration of P was observed in the spongy bone, whereas in men, it was highest in the cartilage. However, overall gender-dependent differences in P concentration were not found to be statistically significant. Subjects were divided into two age groups: 55 to 74 years (HS1) and 75 to 89 years (HS2). There were no statistically significant differences in P concentration between these two age groups. In both age groups, the highest concentration of P was recorded in the spongy bone and the lowest in the infrapatellar fat pad. Of patients undergoing knee joint arthroplasty, 40% were overweight and around 50% were obese. In patients with normal BMI (N) or first degree obesity (O I), the highest P concentration was found in cartilage, while in overweight participants (OW) and those with second or third degree obesity (O II, O III), P was the highest in the spongy bone (Table 2). Overall, P concentration was not found to correlate significantly with BMI.

Table 3 demonstrates the effect of environmental factors—including place of residence, smoking, and alcohol consumption—on P concentrations in knee joint elements. The highest measurements of P concentrations in spongy bone, ACL, and infrapatellar fat pad were taken from patients living in rural areas (V). In cartilage and meniscus, the highest P levels were observed in people living in cities of up to 100,000 inhabitants (SC). However, overall, no significant effect of place of residence on P concentration was observed. Higher P levels in all tissues studied were reported in non-smokers (NS) compared to smokers (S); however, this was not statistically significant. In both groups, the highest concentration of P was found in the spongy bone and the lowest in the infrapatellar fat pad (Table 3). Of the 46 studied, six patients (12.5%) declared alcohol consumption. Comparison of P concentrations between drinking patients (A) and those not consuming alcohol (NA) did not demonstrate statistically significant differences. In alcohol users, the highest concentration of P was observed in cartilage, while in abstinents it was highest in spongy bone. The lowest P concentration in both groups was found in the infrapatellar fat pad (Table 3).

## 4. Discussion

Studies on metal concentration in various types of human bones are carried out in many scientific centers. The majority of research concerns element contents in the femur and ribs. The ribs are made of cartilage tissue and these bones are usually collected posthumously [12,13,14]. Parts of the hip joint are usually taken during arthroplasty surgery, which is commonly performed because of degenerative disease or fractures. However, structures of the knee joint are very rarely used for this type of study.

In existing literature, the documented concentrations of P in various bone elements range from 9400 to 120,000 mg kg^−1^ dw. In this study, the mean concentration of P in spongy tibial bone and cartilage collected from patients from north-western Poland was approximately 70,000 mg kg^−1^ dw. Similar concentrations of this element were found by Zaichick and Zaichick [15] in the iliac crest (~80,000 mg kg^−1^ dw) and Zioła-Frankowska et al. [16] in the spongy femur bone (~60,000 mg kg^−1^ dw). Moriwake et al. [17] noted that average concentrations of P in the lateral and medial meniscus were, respectively, 915 and 1251 mg kg^−1^ dw. In our study, the mean concentration of P in the meniscus was more than three times higher and amounted to 4830.99 mg kg^−1^ dw. The difference may result from the size of the groups and the different study material. The measured concentration of P in the ACL was 10,008.35 mg kg^−1^ dw, much higher than the findings of an earlier study by Tohno et al. [18] in ACL tissues collected from cadavers (685.0 mg kg^−1^ dw).

Gender has demonstrated an influence on the exposure and kinetics of trace elements in the human body due to gender-specific biological differences. Zaichick and Zaichick [15] found a higher concentration of P in the iliac crests of women than men (84.5 and 75.6 mg kg^−1^ dw, respectively). Similarly, Tohno et al. [18] found that P concentrations in the anterior cruciate ligament and femoral head ligament were significantly higher in women (0.81 and 0.56 mg kg^−1^ dw, respectively) than in men (0.60 and 0.46 mg kg^−1^ dw, respectively). Moriwake et al. [17] noted that the concentration of P in the lateral and medial meniscus of women was higher than in men. These differences could be related to the activity of estrogen in the female body, which, in addition to promoting the retention of calcium, potassium, and water in the body, also stimulates the integration of phosphorus into bone tissue [19]. However, in our study, knee joint arthroplasty patients from north-western Poland did not show any influence of gender on P concentration in the studied bone and joint elements.

During aging, the absorption and excretion of certain macro- and microelements is disturbed. Results are inconclusive as to the effect of aging on bodily P levels. Some studies have shown that P concentrations increase with age; for example, Brodziak-Dopierała et al. [20] observed lower P concentrations in the spongy femoral head bone in a group of 59-year-olds than in patients aged 60–69. Conversely, Tohno et al. [18] found a negative correlation between age and P concentration in tendons. An age-related decrease in P concentration has also been described in the aorta, internal thoracic artery, trachea, joint discs, meniscus, and temporomandibular joint [17,21,22,23,24]. In this study, no significant age-dependent difference in P concentration in knee bone and joint elements were found.

Additionally, some studies have found a correlation between increased BMI index and the incidence of osteoarthritis in people over 55 years of age [25]. Elevated body mass impacts not only the hip joint, which is burdened with excessive body weight, but also the knee and ankle joint [26]. Koszowska et al. [27] noted that patients qualified for knee joint arthroplasty generally displayed higher BMIs. As well, micronutrients and macroelements influence numerous metabolic processes and thus play a role in the etiology of obesity with research demonstrating a direct relationship between obesity and deficiencies of certain elements [28,29]. However, in this study, no significant effect of BMI on the concentration of P in knee joint elements was observed.

Geographical location and proximity to industrial plants have been shown to impact the metal content of the human body. Increased quantities of toxic metals in the bodies of inhabitants of highly urbanized areas may lead to synergistic or antagonistic interactions with essential elements, thus affecting body homeostasis and leading to morphological, biochemical, or mechanical changes [30]. However, Dąbrowski [31] showed that the P concentrations in the femoral bone head and neck were similar in inhabitants of urban and rural areas in Wielkopolska. Similarly, Zioła-Frankowska et al. [16] did not find any significant differences in P concentration in compact and spongy femoral bones between patients living in urban and rural areas. Similarly, this study found no significant difference in P concentration in knee joint elements between residents of villages, and small or large cities.

Risk factors for reduced bone mass and osteoarthritis include poor nutrition, low physical activity, smoking, alcohol abuse, and exposure to endocrine disruptors [32]. According to WHO data (2017), tobacco products are consumed by around 1 billion people worldwide [33]. Brodzik-Dopierała et al. [34] showed that the concentration of P in the femoral spongy bone was significantly lower in smokers than in non-smokers. Similarly, Jurkiewicz et al. [35] found that P concentration in the spongy bone of the femoral head of non-smokers was higher than that of smokers. This study, however, found no significant difference in P concentration in knee elements between smokers and non-smokers.

Ethanol exerts an indirect influence on bone cells through its effect on mineral metabolism by hormonal regulation of the vitamin D metabolites parathormone and calcitonin [36]. Many studies have demonstrated the inhibitory effect of alcohol in bone remodeling, mediated by inhibition of vitamin D, which is responsible for equalizing the Ca:P ratio [37]. However, in this study, alcohol consumption showed no significant effects on P concentration in bone and joint elements. Similarly, an earlier study by Zioła-Frankowska et al. [16] did not find any changes in P concentration in bones related to alcohol consumption. It is important to note, however, that results may be affected by the possibility that some patients did not admit to drinking alcohol or smoking for fear of being refused the knee replacement surgery.

## 5. Conclusions

The concentration of phosphorus varies in the structures of the knee joint. Analysis of the relation between biological and environmental factors and phosphorus concentration in the spongy tibial bone, joint cartilage, meniscus, anterior cruciate ligament, and infrapatellar fat pad of knee arthroplasty patients did not find any effect of gender, age, BMI, place of residence, smoking, or alcohol consumption. There is a great need to control the concentration of elements in the human body, because any small change in their concentration may cause the body dysfunction.

## Figures and Tables

**Table 1:** Assessment relationship of P concentration in the majority of knee joint elements

Variables		*R*	*p*
SB	JC	0.025	0.867
	MM	−0.143	0.343
	ACL	0.012	0.939
	HFB	0.293	0.048
JC	SB	0.025	0.867
	MM	−0.386	0.008
	ACL	−0.017	0.911
	HFB	0.026	0.866
MM	SB	−0.143	0.343
	JC	−0.386	0.008
	ACL	0.398	0.006
	HFB	0.038	0.801
ACL	SB	0.012	0.939
	JC	−0.017	0.911
	MM	0.398	0.006
	HFB	0.050	0.740
HFB	SB	0.293	0.048
	JC	0.026	0.866
	MM	0.038	0.801
	ACL	0.050	0.740

Abbreviations: SB, spongy bone; JC, articular cartilage; MM, meniscus; ACL, anterior cruciate ligament; HFB, infrapatellar fat pad, *R*, corrleation, *p*, statistical significance; Notes: The concentration P of elements is expressed in mg kg^−1^ of dry weight.

**Table 2:** Effect of biological factors on phosphorus concentration in knee joint elements (mg kg^−1^ dw) taken from patients from northwestern Poland.

Type of Sample	AM ± SD	Med	Min–Max
Patients (*n* = 46)			
SB	72,746.68 ± 12,061.77	72,471.09	51,266.70–98,448.56
JC	71,554.92 ± 16,157.19	70,799.11	39,067.57–112,679.0
MM	4830.99 ± 7115.91	2476.49	472.85–33,893.78
ACL	10,008.35 ± 16,551.30	4284.61	562.71–90,935.23
HFB	1203.19 ± 1557.07	857.66	235.11–10,325.85
Gender			
Female (*n* = 34)			
SB	73,430.27 ± 11,608.62	72,845.40	52,338.90–97,343.44
JC	69,172.20 ± 15,896.23	69,187.37	39,067.57–112,679.0
MM	4650.83 ± 6297.67	2493.17	472.85–31,308.95
ACL	10,394.87 ± 16,873.36	4284.62	562.71–90,935.23
HFB	1049.90 ± 806.95	876.91	235.11–3470.86
Male (*n* = 12)			
SB	70,809.83 ± 13,614.49	71,663.26	51,266.70–98,448.56
JC	78,305.96 ± 15,576.04	80,738.77	54,499.17–99,729.98
MM	5341.46 ± 9369.19	2358.73	590.48–33,893.78
ACL	8913.19 ± 16,272.24	5175.06	936.93–59,697.62
HFB	1637.52 ± 2772.42	830.82	252.00–10,325.85
Age			
HS 1 (*n* = 26)			
SB	73,530.83 ± 9124.11	73,880.72	936.3–59,697.62
JC	72,094.25 ± 25,533.08	70,988.14	39,067.57–112,679.0
MM	2938.96 ± 2466.80	2469.92	235.11–3470.86
ACL	19,513.81 ± 52,073.33	17,229.73	53,470.49–95,946.84
HFB	749.10 ± 510.90	698.35	485.49–16,776.24
HS 2 (*n* = 20)			
SB	73,882.88 ± 13,065.77	73,171.86	562.71–90,935.23
JC	72,135.13 ± 15,197.24	71,458.87	43,644.2–102,399.15
MM	6313.01 ± 9885.79	2426.79	352.78–10,325.85
ACL	9659.42 ± 19,337.85	5400.29	51,266.70–98,448.56
HFB	1602.49 ± 2180.62	1063.48	472.85–33,893.78
BMI			
Normal weight (*n* = 2)			
SB	75,990.78 ± 20,426.03	75,990.78	61,547.40–90,434.17
JC	93,111.09 ± 13,135.23	93,111.09	83,823.08–102,399.1
MM	1478.90 ± 1404.90	1478.90	485.48–2472.31
ACL	1672.51 ± 915.61	1672.51	1025.07–2319.94
HFB	1042.48 ± 348.32	1042.48	796.18–1288.78
Overweight (*n* = 19)			
SB	74,736.08 ± 12,894.86	73,917.05	52,338.90–98,448.56
JC	71,281.61 ± 15,284.51	71,403.87	43,644.19–99,729.98
MM	4696.37 ± 7052.22	2238.27	590.48–31,308.95
ACL	10,389.42 ± 20,358.44	4127.79	936.93–90,935.23
HFB	1011.19 ± 708.18	886.42	252.00–3227.54
Class I obesity (*n* = 17)			
SB	69,822.70 ± 12,903.61	70,315.04	51,266.70–95,946.84
JC	69,961.24 ± 19,109.26	75,310.84	39,067.57–112,679.0
MM	6869.02 ± 8648.24	4445.61	472.85–33,893.78
ACL	11,414.03 ± 15,049.27	6204.76	995.34–59,697.62
HFB	1426.47 ± 2390.02	759.58	235.11–10,325.85
Class II obesity (*n* = 6)			
SB	71,611.38 ± 3622.87	72,220.95	65,086.30–75,400.92
JC	70,985.20 ± 10,128.26	67,765.47	58,801.38–87,184.72
MM	1675.74 ± 1351.80	1321.33	523.69–4227.50
ACL	4523.12 ± 1981.39	4284.62	2013.50–7345.53
HFB	1397.59 ± 1112.22	1020.02	434.26–3470.86
Class III obesity (*n* = 2)			
SB	78,862.89 ± 9102.20	78,862.89	72,426.66–85,299.12
JC	67,850.65 ± 3314.50	67,850.65	65,506.94–70,194.35
MM	1604.58 ± 1286.16	1604.58	695.13–2514.04
ACL	19,231.35 ± 26,401.45	19,231.35	562.71–37,900.00
HFB	706.77 ± 229.39	706.77	544.57–868.97

Abbreviations: SB, spongy bone; JC, articular cartilage; MM, meniscus; ACL, anterior cruciate ligament; HFB, infrapatellar fat pad; AM, arithmetic average, SD, standard deviation; Med, median; min–max, range; HS 1, age 55–74 years; HS 2, age 75–89 years; BMI, body mass index; Notes: The concentration of elements is expressed in mg kg^−1^ of dry weight. Comparison of P concentration according to age, gender, BMI category is not statistical significance.

**Table 3:** Effect of environmental factors on phosphorus concentration in knee joint elements (mg kg^−1^ dw) taken from patients from northwestern Poland.

Type of Sample	AM±SD	Med	Min–Max
Place of residence			
V (*n* = 5)			
SB	74,641.95 ± 72,515.52	65,086.30	61,363.49–88,945.31
JC	69,368.25 ± 65,506.94	64,303.14	58,801.38–83,818.32
MM	3023.14 ± 2514.04	2480.67	1037.63–4855.88
ACL	10,604.88 ± 4347.22	2652.16	1311.04–37,900.00
HFB	2599.68 ± 868.97	326.85	252.00–10,325.85
SC (*n* = 6)			
SB	69,178.90 ± 67,969.33	53,621.20	52,338.90–98,448.56
JC	78,701.33 ± 79,139.09	57,301.57	39,067.57–112,679.0
MM	7897.08 ± 1895.86	862.09	472.85–31,308.95
ACL	7891.70 ± 6301.34	2319.94	562.71–90,935.23
HFB	1240.97 ± 1266.00	1046.60	235.11–3470.86
C (*n* = 35)			
SB	73,087.54 ± 72,426.66	65,490.03	51,266.70–87,887.98
JC	70,642.20 ± 70,194.35	61,064.08	54,499.17–102,399.1
MM	4563.64 ± 2381.27	836.72	590.48–33,893.78
ACL	10,285.98 ± 4222.01	2511.34	936.93–22,228.15
HFB	997.21 ± 770.13	423.41	886.42–1581.60
Smoking			
Smokers (*n* = 6)			
SB	66,695.90 ± 9966.86	70,315.04	53,470.49–80,069.83
JC	66,318.02 ± 17,913.35	65,962.78	42,754.52–92,812.09
MM	4454.42 ± 5681.36	3265.22	523.68–16,776.24
ACL	3334.95 ± 2661.05	1913.92	995.34–7345.53
HFB	646.54 ± 320.97	759.58	259.44–1141.50
Non-smokers (*n* = 40)			
SB	73,832.71 ± 12,192.07	72,572.29	51,266.70–98,448.56
JC	72,494.87 ± 15,890.83	71,403.87	39,067.57–112,679.0
MM	4898.58 ± 7405.17	2472.31	472.85–33,893.78
ACL	11,206.13 ± 17,709.18	4347.22	562.71–90,935.23
HFB	1303.10 ± 1669.58	886.42	235.11–10,325.85
Alcohol consumption			
A (*n* = 6)			
SB	71,049.86 ± 7679.01	71,365.24	61,399.11–80,069.83
JC	71,579.29 ± 21,700.83	72,405.42	48,694.23–92,812.0
MM	2689.09 ± 1958.69	2317.64	968.24–5152.84
ACL	3537.18 ± 2845.12	3465.53	995.34–6222.34
HFB	760.35 ± 340.19	792.71	314.49–1141.50
NA (*n* = 40)			
SB	72,908.28 ± 12,452.22	72,543.91	51,266.70–98,448.56
JC	71,552.60 ± 15,876.57	70,799.11	39,067.57–112,679.1
MM	5034.99 ± 7403.06	2476.49	472.85–33,893.78
ACL	10,624.65 ± 17,193.17	4284.62	562.71–90,935.23
HFB	1245.36 ± 1622.21	876.91	235.11–10,325.85

Abbreviations: SB, spongy bone; JC, articular cartilage; MM, meniscus; ACL, anterior cruciate ligament; HFB, infrapatellar fat pad; AM, arithmetic average, SD, standard deviation; Med, median; min-max, range; V, patients living in rural areas; SC, people living in cities of up to 100,000 inhabitants; C, people living in cities over 100,000 inhabitants; A, people regularly consuming alcohol; NA, abstainers. Notes: Comparison of P concentration according to place of residence, smoking, alcohol consumption is not statistical significance.
